# The *RAD6*-like Ubiquitin Conjugase Gene *OsUBC7* Has a Positive Role in the Early Cold Stress Tolerance Response of Rice

**DOI:** 10.3390/genes16010066

**Published:** 2025-01-08

**Authors:** Huy Phan, Michael Schläppi

**Affiliations:** Department of Biological Sciences, Marquette University, Milwaukee, WI 53233, USA; huy.phan@marquette.edu

**Keywords:** chilling stress, LTSS, low-temperature stress recovery, *Oryza sativa* L.

## Abstract

Background/Objectives: Cold stress poses a significant threat to Asian rice cultivation, disrupting important physiological processes crucial for seedling establishment and overall plant growth. It is, thus, crucial to elucidate genetic pathways involved in cold stress tolerance response mechanisms. Methods: We mapped *OsUBC7*, a *Radiation-sensitive 6* (*RAD6*)-type homolog of rice, to a low-temperature seedling survivability (LTSS) QTL and used genomics, molecular genetics, and physiological assays to assess its role in plant resilience against low-temperature stress. Results: *OsUBC7* is cold responsive and has higher expression levels in cold-tolerant *japonica* than cold-sensitive *indica*. Overexpression of *OsUBC7* enhances LTSS of *indica* and freezing tolerance of Arabidopsis, increases levels of soluble sugars and chlorophyll A, boosts leaf development after cold exposure, and increases leaf cell numbers and plants size, but it does not affect membrane stability after cold stress exposure. Additionally, *OsUBC7* has a positive role for germinability in the presence of salt and for flowering and yield-related traits. The OsUBC7 protein physically interacts with the developmental stage-specific and histone-modifying E3 ligases OsRFPH2-12 and OsHUB1/2, respectively, and potential target genes such as cell cycle dependent kinases were identified. Conclusions: *OsUBC7* might contribute to cold resilience by regulating sugar metabolism to provide energy for promoting cellular homeostasis restoration after cold stress exposure via new cell growth, particularly in leaf cells crucial for photosynthesis and metabolic activity, possibly by interacting with cell cycle regulating proteins. Overall, the present study suggests that *OsUBC7* may be involved in plant development, reproduction, and stress adaptation, and contributes to a deeper understanding of rice plant cold stress tolerance response mechanisms. *OsUBC7* may be a promising candidate for improving crop productivity and resilience to stressful environments.

## 1. Introduction

Seedling development is crucial for plant growth, establishing the foundation for plant maturation and stress resilience [1,2,3]. It involves physiological changes such as root system establishment and leaf emergence, essential for adaptation to the environment [4,5,6]. This phase influences overall plant architecture and productivity [7,8]. The successful establishment of healthy seedlings is important for ensuring optimal yield and agricultural sustainability, particularly in the case of Asian rice [1,8], a staple food crop supporting billions of people worldwide. Asian rice cultivation involves two primary subspecies, chilling tolerant *japonica* and chilling-sensitive *indica*, each characterized by unique genetic traits and adaptive responses to environmental conditions [1,2,4,9,10].

A common threat to rice cultivation is low-temperature exposure [1,2], which poses a significant challenge to plant growth and development, disrupting various physiological processes essential for seedling establishment and survival [4,11]. Cold stress can induce cellular damage, disrupt membrane integrity, and impair metabolic functions, ultimately compromising plant growth and productivity [1,11,12]. In rice, exposure to low temperatures at early growth stages can lead to reduced germination rates, delayed seedling emergence, and stunted growth, culminating in diminished yields and economic losses for farmers [1,2,9]. The main goal of the present study was advancing our understanding of cold stress tolerance response mechanisms in rice by investigating cold tolerance candidate genes associated with low-temperature seedling survivability (LTSS) quantitative trait loci (QTL) [2,10].

Based on genome-wide association studies (GWAS), we previously identified a multiple low-temperature stress tolerance trait QTL, *qMT7-3*, containing the most significant single nucleotide polymorphism (SNP), or peak SNP, within the leader sequence of LOC_Os07g07240, coding for the E2 type ubiquitin conjugase gene *OsUBC7* with strong similarity to yeast *Radiation-sensitive 6* (*RAD6*) [2]. Previous work has shown that *RAD6* is involved in critical cellular processes such as DNA repair, cell cycle regulation, gene expression regulation, and protein quality control mechanisms [13,14,15,16,17,18], and that *OsUBC7* is associated with yield-related traits and stress tolerance mechanisms [19]. We hypothesized that in addition to those processes, *OsUBC7* is also involved in abiotic stress response pathways by interacting with different protein partners that help promote resilience to low-temperature stress via protein ubiquitination. This is supported by previous studies showing that protein ubiquitination is involved in abiotic stress tolerance response mechanisms [20,21,22,23,24,25], and by metadata analyses showing that *OsUBC7* is differentially expressed under various environmental conditions and in response to changing environmental cues, including temperature stress [15,19]. While most previous work has focused on E3 type ubiquitin ligases, a recent study has shown that upregulation of the E2 encoding gene *OsUBC12* has a positive effect on rice cold tolerance [26].

In the present study, we investigated the potential regulatory role of *OsUBC7* in cold stress tolerance response mechanisms of rice by using genomics, reverse genetics, and physiological approaches. We determined that similar to *OsUBC12*, upregulation of *OsUBC7* has a positive effect on rice cold temperature stress resilience by affecting various metabolic and physiological processes such as soluble sugar accumulation, chlorophyll levels, growth rate, plant size, and yield-related traits. Moreover, we showed that *OsUBC7* has a positive effect on salt tolerance and that it interacts with different RING-finger containing E3 ubiquitin ligases and protein partners, indicating that it may affect different aspects of seedling growth and development and abiotic stress adaptation. This knowledge could be used to devise future breeding strategies to enhance crop productivity and resilience in the face of a changing environment.

## 2. Materials and Methods

### 2.1. Plant Materials and Growth Condition

Rice seedlings were grown hydroponically in an AR-66L growth chamber (Percival Scientific, Perry, IA, USA) under a photon flux of ~165 μE and 12/12 h light/dark cycles at 28/25 °C day/night temperatures. On the tenth day, water was replaced with quarter-strength Murashige and Skoog (MS) liquid medium. Arabidopsis seeds, both wild-type (WT) and transgenic, were surface-sterilized using 10% sodium hypochlorite before germination on agar-solidified half-strength MS medium in an AR-66L growth chamber under standard conditions of 16/8 h light/dark cycles and 22/20 °C day/night temperatures at a photon flux of ~165 μE. The *aus* subgroup reference accession Kasalath from the *indica* rice subspecies and Arabidopsis ecotype Columbia-0 (Col-0) were used for the generation of overexpression (OE) transgenic lines, while the *AtUBC2* knockout (KO) line SALK_152462C was obtained from the Arabidopsis Biological Resource Center at Ohio State University.

### 2.2. Cold-Temperature Stress Treatment of Rice Plants

Rice seeds were germinated for 2 days at 37 °C and grown as described in Section 2.1. For most experiments, two-week-old lines overexpressing *OsUBC7* from the strong 35S enhancer-mannopine synthase promoter (cloning primers and degree of upregulation shown in Supplementary Tables S1 and S2, respectively) and WT Kasalath seedlings were continuously stressed at 10 °C for 2 days, as this treatment led to half plant population survival (LT50 = 50% survival) for Kasalath WT plants [4,11]. All physiological assays on rice seedlings were performed at the conclusion of the cold-temperature treatment. For survival scoring, cold-treated rice seedlings were returned to standard growth conditions in growth chambers as described in Section 2.1 for one week of recovery growth.

### 2.3. Assessment of Percent Low-Temperature Seedling Survivability (LTSS) in Rice

Two-week-old rice seedlings were subjected for various periods of time to 10 °C cold-temperature stress and then permitted to recover for 7 days under 12/12 h photoperiod conditions at 28/25 °C (day/night) with a photon flux of ~165 μE. Mean survivability was determined as percent LTSS after 7 days of recovery, calculated by dividing the number of green and healthy-looking seedlings after recovery by the initial number of healthy-looking seedlings before cold treatment × 100.

### 2.4. Assessment of Freezing Stress Tolerance in Arabidopsis

Col-0 WT and *OsUBC7* OE transgenic lines were exposed to freezing stress using a refrigerated circulating bath (Polyscience, Niles, IL, USA). Two-week-old individual seedlings were carefully removed from agar plates and transferred onto pre-wetted Light-Duty Tissue Wipes (VWR, Radnor, PA, USA) in 50 mL conical tubes, ensuring humidity was maintained by covering them with additional pre-wetted Tissue Wipes. The tubes were then placed in the circulating bath at −2 °C for 15 min, followed by the addition of approximately 50 μL of double distilled (dd) H_2_O ice chips to facilitate ice nucleation in the plant tissues. After incubating at −3 °C for 75 min, the seedlings were allowed to thaw inside the tubes at room temperature for 30 min before being transferred to pre-wetted filter papers. Subsequently, seedlings were kept under standard growth conditions in the AR-66L growth chamber (16/8 h light/dark cycles and 22/20 °C day/night temperatures). Percent freezing survival was calculated at 4, 5, 6, and 7 days of recovery growth by dividing the number of green and healthy-looking seedlings by the initial number of healthy-looking seedlings before treatment × 100.

### 2.5. Gene Expression Analysis

Quantitative reverse-transcription PCR (qPCR) analyses were conducted to assess mRNA abundance in rice plants. Following various treatments, whole seedling or leaf tissues at the flowering stage were collected and promptly frozen in liquid nitrogen. Total RNA was extracted using the Trizol reagent (Invitrogen, Carlsbad, CA, USA). Subsequently, first-strand cDNA was synthesized utilizing M-MLV reverse-transcriptase (Promega, Madison, WI, USA), and qPCR reactions were performed using 2× universal SYBR green supermix (BioRad, Hercules, CA, USA) in a BioRad CFX96 Touch™ Real-Time PCR Detection System under the following conditions: initial denaturation at 95 °C for 5 min, followed by 40 cycles of denaturation at 95 °C for 15 s, annealing at 55 °C for 30 s, and extension at 72 °C for 1 min. The primer sequences used are shown in Supplementary Table S1.

### 2.6. Measurement of Sugar Content in Two-Week-Old Rice and Arabidopsis Seedlings

Sugar content in plant tissues was assessed by a two-step process involving sugar extraction and colorimetric staining, as previously described in [27,28], with minor modifications. Collected tissues were first ground in liquid nitrogen, weighed, and treated on ice with 1 mL of 12:5:3 methanol/chloroform/water (MCW) extraction buffer containing lactose (0.1 μg·μL^−1^). After brief mixing and incubation at 50 °C for 30 min, samples were centrifuged (12,052× *g*) for 5 min at room temperature, and the resulting supernatant was transferred to new conical tubes and kept on ice. Subsequently, another round of centrifugation was performed after adding 1 mL of MCW extraction buffer without lactose to the plant tissues, and the supernatant was collected and transferred as before. This process was repeated, with sterile ddH_2_O added to each tube as the third and final step. After mixing and centrifugation, 1.5 mL of the aqueous solution containing the sugar extract was transferred to microcentrifuge tubes and stored at −20 °C overnight. For the colorimetric assay, 40 μL of each sample was combined with 200 μL of 5% phenol and 1 mL of concentrated sulfuric acid, followed by incubation at room temperature for 20 min. Absorbance of the samples was then measured at 510 nm, and a standard curve was generated using a 1 mg·mL^−1^ sucrose stock solution. The results were normalized by fresh tissue weight (mg). This experiment was conducted three times, with nine rice plant samples collected each time, where every three plants were pooled together. For Arabidopsis, thirty plant samples were collected, with every ten plants pooled together for each reading.

### 2.7. Measurement of Percent Electrolyte Leakage (EL)

Following the low-temperature stress treatments, 4 equally sized tissue sections from the middle part of the second leaf were collected from rice seedlings, while whole plants of Arabidopsis were collected. Tissue samples were washed in ddH_2_O after which they were transferred into screw-cap glass tubes containing 5 mL of ddH_2_O and placed on a rotary shaker at 200 rpm for 1 h to facilitate the release of electrolytes from damaged cells. Electrolyte conductivity of the 4 replicate samples was measured in microsiemens (µS) twice using a hand-held LAQUAtwin B-771 conductivity meter (Horiba Scientific, Kyoto, Japan), with precautions taken to ensure glass tube cleanliness and close to 0 µS conductivity of the ddH_2_O supply. Subsequently, after the initial measurement, tissue samples were boiled for 10 min to release the total amount cellular electrolytes. Following cooling to room temperature, samples were again shaken at 200 rpm for 1 h, and total conductivity was measured. Electrolyte leakage (EL) for each treatment was then calculated as %EL = [(initial conductivity)/(total conductivity)] × 100.

### 2.8. Measurement of Malondialdehyde Concentration

The extent of chilling-induced lipid peroxidation in *OsUBC7* OE and wild-type rice lines was evaluated using a spectrophotometric assay to measure thiobarbituric acid reactive substances (TBARS), an estimate of malondialdehyde levels [29]. Frozen aerial tissues from each line, prepared in triplicate, were ground to a fine powder in liquid nitrogen and stored in 1.5 mL tubes. For each sample, 100 mg was analyzed in triplicate according to the TBARS assay protocol, adapted from Hodges et al. [30] and modified by Landi [31]. This modified assay detects the thiobarbituric acid–malondialdehyde (TBA-MDA) complex, which forms when MDA in tissue extracts reacts with TBA. The TBA-MDA complex absorbs light at 532 nm, allowing for precise spectrophotometric quantification in rice tissue extracts. Protocol adjustments by [31] minimize absorbance interference from compounds with similar spectral properties, thus, reducing the risk of overestimating MDA levels due to non-chilling-related stress factors.

### 2.9. Measurement of Chlorophyll Content in Rice Flag Leaves

Chlorophyll content of Kasalath flag leaves at the flowering stage was determined as previously described [32], with minor modifications. Flag leaf tissues were submerged in 7 mL of 100% ethanol and kept in the dark at room temperature for 4 days. Subsequently, leaves were removed from ethanol and dried at 60 °C for 24 h to determine dry weight. The absorbance of 1 mL of the ethanol was measured, using a spectrophotometer, at 665 nm and 649 nm to quantify chlorophyll A and B, respectively, with 1 mL of pure ethanol serving as blank. Chlorophyll content was calculated as follows: chlorophyll A, (13.70 × A665 nm − 5.76 × A649 nm) μg·mL^−1^; chlorophyll B, (−7.60 × A665 + 25.8 × A649) μg·mL^−1^. All values were multiplied by 7 to account for the initial 7 mL of ethanol used. The amounts of chlorophyll A and B were normalized by the milligrams of dry weight of leaf tissues. This experiment was conducted three times, with at least six plant samples collected each time.

### 2.10. Measurement of Leaf Length, Leaf Width, and Yield-Related Phenotypes

The flag leaf dimensions were assessed using a 30 cm ruler, with precise measurements recorded to the nearest mm. Half-length of the flag leaf was determined by dividing total length by 2. Quantification of seed yield in both *OsUBC7* OE and WT plants was performed by counting seeds produced by representative plants from each line across multiple transgenic (T) generations (T_2_, second; T_3_, third, and T_4_, fourth). The number of seeds per panicle was calculated by dividing the total seed yield by the corresponding total number of panicles per plant. Percent fertility was calculated by dividing the number of filled grains by the total number of filled and unfilled grains ×100.

### 2.11. Yeast Two-Hybrid (Y2H) Analyses

The coding sequence of *OsUBC7* was PCR amplified using cDNA derived from the *temperate japonica* accession Krasnodarskij 3352 as a template with forward primer 5′-AAACATATGATGTCGACGCCGGCGAGGAA-3′ (*Nde*I site underlined) and reverse primer 5′-AAAGGATCCCTAGTCAGCTGTCCAGCTCT-3′ (*Bam*HI site underlined) and cloned into pGEM-T-easy (Promega). *OsUBC7* cDNA was removed from the vector via NdeI and BamHI restriction enzyme digestion and cloned into the pGBKT7 bait vector (TakaraBio, San Jose, CA, USA). The coding sequence of the E3 ubiquitin ligase gene *OsRFPH2-12* (LOC_Os01g58780) was amplified from the same cDNA source using forward primer 5′-AAACATATGATGCCAAATAGAGCCACGCAT-3′ (*Nde*I site underlined) and reverse primer 5′-AAAGGATCCTCAGTAGTCGTAATGCCACTC-3′ (*Bam*HI site underlined) and cloned into pGEM-T-easy. The RING finger domain of the E3 ubiquitin ligase genes *OsHUB1*/*OsHUB2* (LOC_Os04g46450/LOC_Os10g41590) were PCR amplified using the same cDNA source as template with forward primer 5′-AAAGAATTCATGGAGGGTGTTAAAGCAAA-3′ (*Eco*RI site underlined) and reverse primer 5′-AAAGGATCCTCATATGTAGATAGGTTTCA-3′ (*Bam*HI site underlined) and cloned into pGEM-T-easy. E3 ligase gene sequences were removed from the pGEM-T-easy vector via NdeI and BamHI and EcoRI and BamHI digestions for *OsRFPH2-12* and *OsHUB1*/*OsHUB2* cDNA, respectively, and cloned into the pGAKT7 prey vector (TakaraBio). Positive protein interaction control vectors pGBKT7-53 (bait) and pGADT7-T (prey) were obtained from TakaraBio. pGBKT7-OsUBC7 (bait) and pGAKT7 (empty prey), and pGBKT7 (empty bait) and pGAKT7-OsRFPH2-12 (prey) and pGAKT7-OsHUB1/OsHUB2 (prey) were used as negative controls. Each interacting pair of plasmids was transformed into yeast strain Y2HGold (TakaraBio) using the heat shock method. Yeast cultures were grown overnight at 30 °C in YPD, after which 1 mL of the culture was diluted into 5 mL of fresh YPD and incubated at 30 °C for 3–5 h until the culture reached an OD_600_ of 0.2–0.8. Isolated vector DNA (5.0 µL) was added to sterile Eppendorf tubes, and for each sample a master mix consisting of 100 µL One Step Buffer, 10 µL 1M DTT, and 5 µL salmon sperm DNA was prepared. Yeast cultures were centrifuged at 3000 rpm for 2 min, resuspended in 1 mL ddH_2_O, and centrifuged again at maximum speed for 30 s. The pellet was resuspended in 115 µL of master mix, and 115 µL of this mixture was added to the DNA vector-containing tubes. After brief mixing, samples were heat shocked at 45 °C for 30 min. Cells were plated onto synthetically defined (SD) selective medium with the following “Dropped Out” formulations: SD/–Leu/–Trp Double Dropout (DDO) for selection of the bait and prey vectors, and SD/–Leu/–Trp/–His/–Ade Quadruple Dropout (QDO) for selection of the two vectors and protein–protein interaction between the bait and prey construct. Yeast colonies were obtained after 3 days of incubation at 30 °C.

### 2.12. Assessment of Salt Stress Tolerance in Arabidopsis

Salt germination assays were performed as described previously [11,32]. A total of 100 surface-sterilized seeds of the Col-0 WT and one *OsUBC7* OE homozygous transgenic Arabidopsis line were arranged in a grid pattern on agar-solidified half-strength MS medium supplemented with various NaCl concentrations (0, 50, and 100 mM). The seeds were allowed to germinate for two weeks under standard growth conditions. Each plate contained ~50 seeds from WT and OE lines, with seed positions randomized across three plates to mitigate potential positional effects. Germination, defined as the emergence of the radicle, was monitored daily during a two-week period. Percent germination for each line was calculated for each replicate plate as % germination = [(number of germinated seeds)/(total number of seeds)] × 100. Mean germination percentages were calculated from replicate experiments.

### 2.13. Statistical Analyses

Phenotypic differences were primarily evaluated using Welch’s ANOVA with OriginPro2018 (Origin Lab Corporation, Northampton, MA, USA). For yield-related traits, the Games–Howell non-parametric post hoc test was applied manually in Microsoft Excel to account for unequal sample sizes, deviations from normality, and variance inconsistencies. This approach allowed proper analysis between overexpression and wild-type lines.

## 3. Results

### 3.1. Identification of LOC_Os07g07240 as a Chilling Tolerance Candidate Gene Associated with qMT7-3

**Figure 1:**
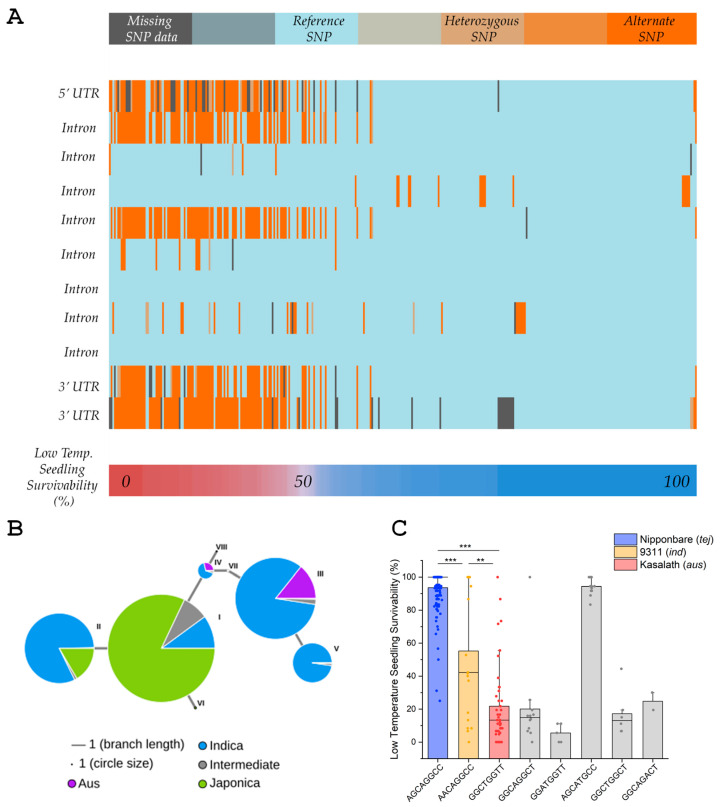
Single-nucleotide polymorphisms (SNPs) and haplotypes of *OsUBC7*. (**A**) 11 SNPs of *OsUBC7* in the 354 accessions of the Rice Diversity Panel 1 correlate with low-temperature seedling survivability (LTSS) scores. Two-week-old seedlings of the 354 accessions were exposed to constant 10 °C for 7 days and allowed to recover at warm temperatures for 7 days (28/25 °C day/night), after which LTSS was determined. (**B**) Haplotype analysis of *OsUBC7* using RiceVarMap [33] data from a population of 4402 rice accessions. (**C**) Haplotype–LTSS correlation analysis based on three major haplotypes of mentioned accessions. *p* values for Two-Way ANOVA: (**) *p* < 0.01; (***) *p* < 0.001.

Using the Rice Diversity Panel 1 (RDP1) from the USDA and a genome-wide association study (GWAS) mapping approach, we previously identified numerous Multi-cold tolerance response Trait (MT) quantitative trait loci (QTL) [2]. Since the peak QTL single nucleotide polymorphism (SNP) within the 3.4–3.7 Mb QTL region for *qMT7-3* on chromosome 7 was in the transcribed region of LOC_Os07g07240, annotated as a Rad6-type ubiquitin-conjugating (UBC) enzyme encoding a gene classified as *OsUBC7*, we focused on this gene as a candidate for *qMT7-3*. In silico genomics analyses indicated that *OsUBC7* met several criteria for a cold tolerance candidate gene, including differential gene expression between chilling-tolerant *temperate japonica* Nipponbare and chilling-sensitive *indica* line 93-11 in response to low-temperature treatment, and the presence of additional 10 SNPs in the coding regions between the rice reference genomes of two *temperate japonica* lines, Nipponbare and Krasnodarskij 3352, and the two *aus* lines, Carolino 164 and Kasalath. Of the eleven SNPs, one (the peak QTL SNP) was in the 5′ untranslated region (UTR), eight were in introns, and two in the 3′ UTR. Matching of these SNPs in all 354 accessions of the Rice Diversity Panel 1 (RDP1) used for the GWAS [2] to low-temperature seedling survivability (LTSS) scores showed that accessions with low LTSS scores predominantly had *indica* type SNPs at the 5′ UTR, at three intron locations, and both 3′ UTR regions, which we defined as an “alternate” haplotype compared to the *japonica* reference genome haplotype (Figure 1A). Further analyses uncovered eight different haplotypes (I, II, III, IV, V, VI, VII, VIII) among 4000 accessions analyzed. Among those, three major haplotypes (I, II, III) covered more than 1000 accessions. Haplotype I (AGCAGGCC) was predominantly found in *japonica* accessions such as Nipponbare (82.11%), haplotype II (AACAGGCC) mostly in *indica* accessions such as 93-11 (81.92%) and some *japonica* accession (16.52%), and haplotype III (GGCTGGTT) predominantly in *indica* (83.49%) and *aus* accessions such as Kasalath (14.17%) (Figure 1B). Matching these haplotypes to LTSS scores of the 354 RDP1 accessions showed that accessions with haplotype I had high LTSS scores, those with haplotype II intermediate LTSS scores, and those with haplotype III low LTSS scores (Figure 1C).

Taken together, these data suggested that *OsUBC7* is a probable LTSS trait candidate gene in rice, supported by differential expression patterns and SNP differences between chilling-tolerant and chilling-sensitive accessions, pointing to a potential role in mediating cold stress tolerance responses. The significant correlation between *OsUBC7* haplotypes and LTSS scores (Figure 1C) further emphasized its potential role in modulating cold stress tolerance in rice, highlighting the importance of studying the gene further for future research strategies to enhance rice crop resilience against cold temperature stress.

### 3.2. OsUBC7 Is Upregulated by Low-Temperature Exposure

**Figure 2:**
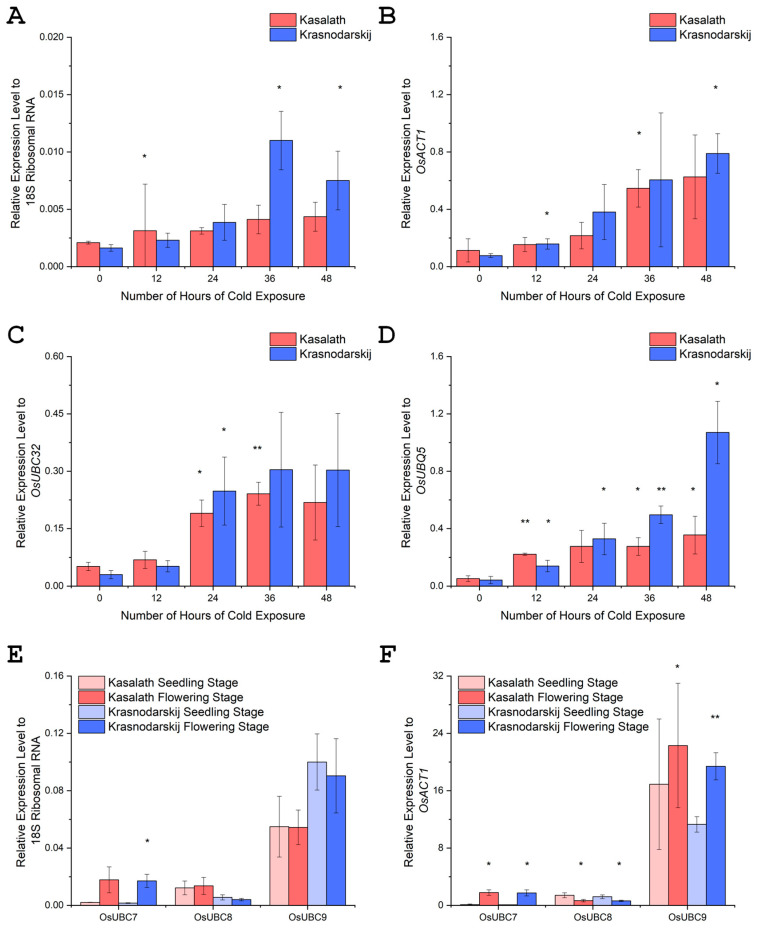
Gene expression of *OsUBC7* and its paralogs in different rice accessions and different temperature conditions. (**A**–**D**) Time series of *OsUBC7* mRNA abundance in 2-week-old seedlings under warm control and low-temperature exposure (10 °C for *aus* Kasalath, 4 °C for *temperate japonica* Krasnodarskij 3352). Four housekeeping genes, 18S ribosomal RNA, *OsACT1*, *OsUBC32*, and *OsUBQ5*, corresponding to panels **A**, **B**, **C**, and **D**, respectively, were used for normalization. (**E**,**F**) mRNA abundance of *OsUBC7*, *OsUBC8*, and *OsUBC9* in leaf tissues of Kasalath and Krasnodarskij 3352 at the 2-week-old seedling stage and in flag leaves at the flowering stage. 18S ribosomal RNA (panel **E**) and *OsACT1* (panel **F**) were used for normalization. (*) *p* ≤ 0.05, (**) *p* ≤ 0.01 (Welch’s *t*-test).

To validate that *OsUBC7* was cold regulated, we measured mRNA abundance in both *indica* and *japonica* subspecies under warm control conditions and after 12, 24, 36, and 48 h of exposure to 10 °C (for *indica*) and 4 °C (for *japonica*) using four different housekeeping genes to normalize expression levels: 18S ribosomal RNA, *OsACT1*, *OsUBC32*, and *OsUBQ5*. Although normalizing using different housekeeping genes gave slightly different results, overall, *OsUBC7* mRNA increased progressively in both *indica* and *japonica* accessions during cold temperature exposure (Figure 2A–D; Supplementary Table S3). Compared to warm controls, fold changes in OsUBC7 mRNA levels in the cold were higher in *japonica* than in *indica* samples. When averaging values using different housekeeping genes, log2 fold changes in OsUBC7 mRNA abundance at 12 h of cold were 0.88 ± 0.81 and 1.01 ± 0.53 in *indica* and *japonica*, respectively; at 24 h of cold, 1.45 ± 0.84 and 2.39 ± 0.83 in *indica* and *japonica*, respectively (paired *t*-test, *p* < 0.05); at 36 h of cold, 1.97 ± 0.67 and 3.15 ± 0.36 in *indica* and *japonica*, respectively (*p* < 0.05); and at 48 h, 2.09 ± 0.75 and 3.39 ± 1.00 fold in *indica* and *japonica*, respectively (*p* < 0.05).

Taken together, these results indicated that *OsUBC7* mRNA levels are upregulated by cold temperature exposure in both the *indica* and *japonica* subspecies of rice. Across different time points and normalization methods, the log2 fold change in *OsUBC7* expression consistently showed upregulation in response to low temperatures, with *japonica* showing significantly higher mean log2 fold changes than *indica* after 24–48 h of cold exposure. Overall, this progressive upregulation of *OsUBC7* in response to cold suggests that the gene might play a role in the cold stress tolerance response mechanisms of rice.

### 3.3. Overexpression of OsUBC7 Enhances LTSS Scores of Transgenic Rice Lines

**Figure 3:**
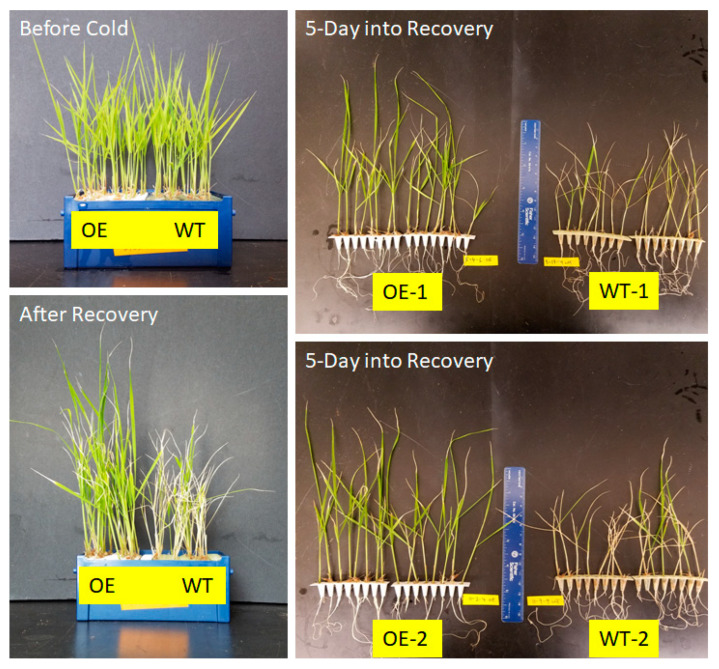
Phenotype of *OsUBC7* overexpression (OE) rice lines and *aus* Kasalath wild-type (WT) plants. (**Top left**) Two-week-old seedlings of *OsUBC7* OE lines and WT plants before cold treatment. (**Bottom left**) Two-week-old seedlings of *OsUBC7* OE lines and WT plants after cold treatment and 4 days of recovery. (**Top right**) *OsUBC7* OE-1 line and segregated WT-1 plants after 5 days of recovery from cold treatment. (**Bottom right**) *OsUBC7* OE line OE-2 and segregated WT-2 plants after 5 days of recovery from cold treatment.

**Figure 4:**
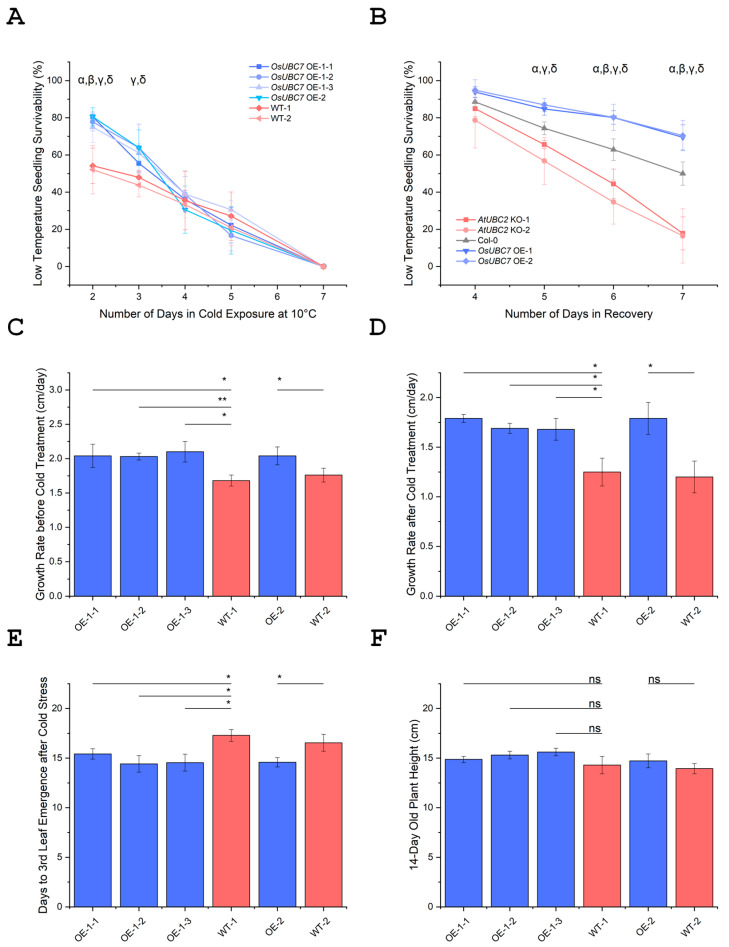
Cold stress-related physiological phenotypes of *OsUBC7* overexpression (OE) transgenic lines and wild-type (WT) plants. (**A**) Low-temperature seedling survivability (LTSS) of *OsUBC7* OE and WT rice plants. (**B**) LTSS of *OsUBC7* OE Arabidopsis, Col-0 WT, and *AtUBC2* (*OsUBC7* homolog) knockout (KO) plants exposed to −3 °C and recovering over a 7-day period. (**C**) Growth rate before cold treatment of *OsUBC7* OE and WT rice plants. (**D**) Growth rate after cold treatment of *OsUBC7* OE and WT rice plants. (**E**) Days to third leaf emergence after cold stress. (**F**) Plant height of 14-day-old *OsUBC7* OE and WT seedlings. (α, ß, γ, δ) significant difference in OE or *AtUBC2* KO lines compared to their respective WT plants. *p* values for Two-Way ANOVA: (*) *p* < 0.05; (**) *p* < 0.01; (ns) no significance.

To determine whether constitutive overexpression (OE) of *OsUBC7* in cold-sensitive rice accessions had a positive effect on LTSS, fourth generation (T_4_) OE transgenic plants were exposed for 2, 3, 4, 5, and 7 days to 10 °C and allowed to recover for one week at warm temperatures. After 2 days of cold, all four OE lines tested had significantly higher LTSS scores compared to segregated wild-type (WT) lines (*p* < 0.05). After 3 days, two OE lines had significantly higher LTSS scores than segregated WT plants (*p* < 0.05), while the other two had a non-significant trend. After 4 and 5 days of cold stress, both transgenic and WT lines had similar LTSS scores, and after 7 days, all lines reached an LTSS score of zero (Figure 3 and Figure 4A).

These results indicated that OE of *OsUBC7* in a cold-sensitive rice accession helped overall seedling survival of plants exposed to 10 °C for a short period of 2–3 days, increasing LTSS scores by approximately 50%. After longer exposure times, LTSS scores between *OsUBC7* OE transgenic and WT lines were similar, suggesting that *OsUBC7* might be an early cold stress tolerance response gene.

### 3.4. Overexpression of OsUBC7 Increases Freezing Tolerance of Transgenic Arabidopsis Lines

Since OE of *OsUBC7* enhanced LTSS scores of transgenic rice lines after cold exposure, we overexpressed *OsUBC7* in Arabidopsis to determine whether it had a positive effect on freezing survival in a heterologous system sharing a closely related homolog. The results indicated that over a 7-day recovery period, OE lines consistently had higher freezing survival than Col-0 WT and *AtUBC2* (the Arabidopsis homolog of *OsUBC7*) knockout (KO) lines after a 75 min exposure to −3 °C. (Figure 4B; Supplementary Figure S1A–D). Taken together, we observed a consistent enhancement of LTSS across different plant species, from the monocot rice to the eudicot Arabidopsis, highlighting potentially a general role of RAD6-type ubiquitin-conjugating enzyme encoding genes in enhancing resilience of plants against short periods of low-temperature stress.

### 3.5. OsUBC7 OE Promotes Plumule Recovery Growth and Enhances Formation of the Third Leaf After Low-Temperature Exposure

As shown previously, rice lines can have relatively good LTSS values but fail to resume growth during the recovery period at higher temperatures [9]. Seedlings can remain green, indicating that the leaves are chilling-tolerant, but cell division and elongation activity at the base of leaves or in the shoot apical meristem is compromised after the cold treatment. Because *OsUBC7* encodes a potential RAD6-type checkpoint protein for the cell cycle, we investigated whether OE of this gene had a positive effect on plumule recovery growth as a potential mechanism for why transgenic lines had higher LTSS scores than WT plants after cold stress. OE lines had indeed a faster plumule growth rate than WT plants (Figure 4D; *p* < 0.05). Additionally, all four OE lines developed their third leaves earlier than WT plants (Figure 4E; *p* < 0.05).

These results showed that OE in young rice seedlings of the potential cell cycle regulatory protein-encoding gene *OsUBC7* significantly enhanced plumule recovery growth after cold exposure, promoting a tendency for taller OE than WT seedlings (Figure 4F). Moreover, the third leaf emerged consistently earlier in OE than WT lines, suggesting a potential role of *OsUBC7* in promoting faster recovery and growth rates to replace damaged cells in response to cold stress.

### 3.6. Overexpression of OsUBC7 in Rice and Arabidopsis Increases Soluble Sugar Content Under Warm, Low-Temperature Exposure, and Recovery Conditions

**Figure 5:**
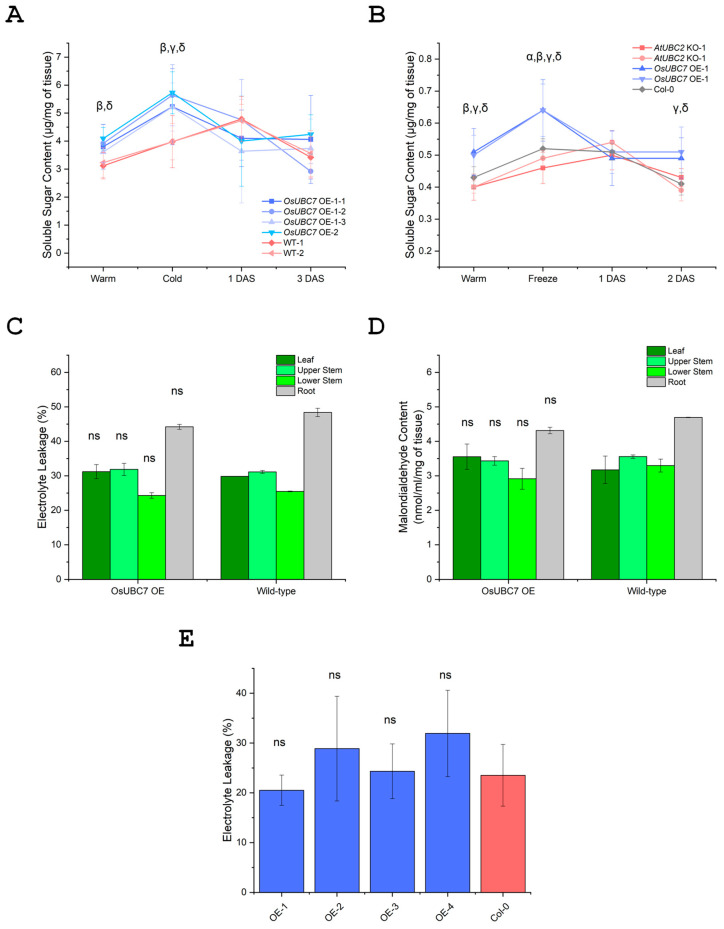
Effect of *OsUBC7* overexpression (OE) in transgenic plants on cold stress-related metabolites. (**A**) Soluble sugar content in *OsUBC7* OE rice lines and wild-type (WT) plants. (**B**) Soluble sugar content in *OsUBC7* OE Arabidopsis lines, WT Col-0, and *AtUBC2* knockout (KO) plants. (**C**) Mean % electrolyte leakage levels in different tissues of *OsUBC7* OE rice lines and WT plants after cold exposure. (**D**) Malondialdehyde content in different tissues of *OsUBC7* OE rice lines and WT plants after cold exposure. (**E**) Electrolyte leakage in *OsUBC7* OE Arabidopsis lines and WT plants. (α, ß, γ, δ) significance detected in *OsUBC7* OE transgenic or *AtUBC2* KO lines compared to their corresponding WT lines. *p* < 0.05, Two-Way ANOVA. DAS, day(s) after stress; ns, not significant.

Because *OsUBC7* OE transgenic lines had improved recovery after cold stress in rice and freezing stress in Arabidopsis, we determined whether OE lines had a higher amount of soluble sugars than WT plants as a potential energy source for successful recovery. Under warm conditions, *OsUBC7* OE rice lines had higher soluble sugar contents than WT plants, with significantly higher levels in OE-1-2 and OE-2 (*p* < 0.05). After 2 days of cold exposure at 10 °C, all three OE lines had significantly higher levels of soluble sugar than WT plants (*p* < 0.05), while during recovery at warm temperatures, soluble sugar contents in OE lines were like those in WT plants (Figure 5A).

Two *OsUBC7* OE Arabidopsis lines also had significantly higher soluble sugar contents than Col-0 WT and *AtUBC2* KO plants (*p* < 0.05). Following freezing treatment, OE lines maintained higher soluble sugar contents than WT plants and KO lines (*p* < 0.05). After 2 days of stress recovery, *OsUBC7* OE lines retained higher soluble sugar levels than WT and KO lines (Figure 5B).

These results indicated that *OsUBC7* OE transgenic lines have higher levels of soluble sugars than WT plants under both normal and low-temperature conditions, potentially contributing to their improved recovery after cold stress and freezing stress. Hence, *OsUBC7* may have a potential role in sugar metabolism regulation, which could be a key factor in enhancing cold stress tolerance, in both rice and Arabidopsis, and possibly, other plant species.

### 3.7. Overexpression of OsUBC7 Does Not Improve Membrane Damage After Low-Temperature Exposure

Since *OsUBC7* OE transgenic lines had better survival rates than WT plants following cold or freezing stress, we investigated whether this improvement correlated with enhanced membrane stability right after stress exposure. To assess membrane stability, electrolyte leakage (EL) from various plant parts was measured, including the second leaf, upper stem, lower stem, and roots for rice, and whole plants for Arabidopsis. In rice, EL levels were similar in *OsUBC7* OE lines and WT plants in all plant tissues tested, leaves, upper stems, lower stems, and roots (Figure 5C). Similarly, *OsUBC7* OE Arabidopsis lines had similar EL levels as Col-0 WT plants after freezing treatment (Figure 5E). These results indicated that OE of *OsUBC7* does not positively affect membrane integrity in these plants, although it might have a small but non-significant positive effect in rice roots. This indicated that the positive effect on LTSS seen in *OsUBC7* OE lines is not due to protection of membrane integrity during cold stress exposure, at least not as measured by the electrolyte leakage assay.

### 3.8. Overexpression of OsUBC7 Does Not Result in Lower Lipid Peroxidation Levels After Low-Temperature Exposure

To evaluate an alternative potential positive effect of *OsUBC7* OE on membrane stability, we measured malondialdehyde levels as a final product of polyunsaturated fatty acid peroxidation due to excess of reactive oxygen species (ROS) in cells extracted from different parts of rice plants, including the second leaf, upper stem, lower stem, and root. This showed that malondialdehyde levels were similar in OE and WT lines across different plant parts (Figure 5D). Although *OsUBC7* OE lines had a trend for slightly lower malondialdehyde levels than WT plants in sections other than leaves, the differences were not significant. This indicated that OE of *OsUBC7* may somewhat help to reduce the degree of lipid peroxidation in roots and lower stem regions after cold stress exposure, although the effect as measured by malondialdehyde levels was not significant.

### 3.9. Leaves of OsUBC7 Overexpression Lines Have a Higher Cell Density than Wild-Type Plants

**Figure 6:**
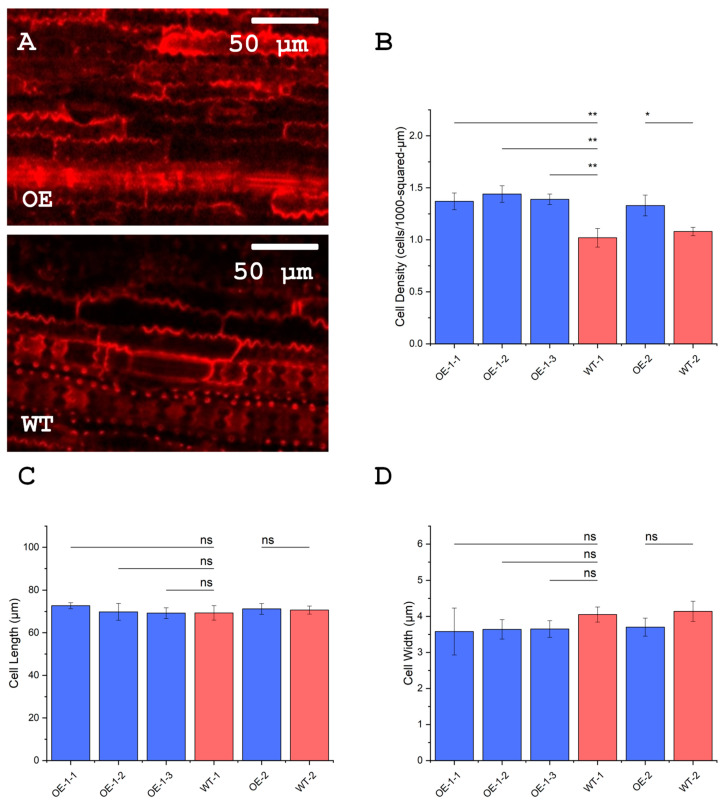
Effect of *OsUBC7* overexpression (OE) on leaf cell dimensions and cell density. (**A**) Leaf tissue of *OsUBC7* OE and segregated wild-type (WT) lines stained with propidium iodide. (**B**) Cell density of *OsUBC7* OE and WT lines. (**C**) Cell lengths of *OsUBC7* OE and WT lines. (**D**) Cell widths of *OsUBC7* OE and WT lines. *p*-values for Two-Way ANOVA: (*) < 0.05; (**) < 0.01; (ns) no significance.

Because OE of *OsUBC7* boosted plumule growth rates and showed a trend toward increased plant height at the seedling stage, we investigated whether this resulted from an increase in cell elongation or an increase in cell number. We found that all four OE lines had a higher leaf cell density than segregated WT plants (Figure 6A,B; *p* < 0.05). By contrast, *OsUBC7* OE lines and WT plants had similar leaf cell lengths (Figure 6C). Interestingly, all four *OsUBC7* OE lines showed a trend for narrower leaf cells than WT plants (Figure 6D).

### 3.10. Compared to Other RAD6 Paralogs, OsUBC7 Is Highly Expressed at the Flowering Stage

Because public databases indicated that *OsUBC7* expression was highest during the flowering stage, we analyzed mRNA levels of *OsUBC7* in leaf tissues of 2-week-old seedlings and leaf tissues of plants that were at the flowering stage (3 days after emergence of the first flag leaf). Normalized gene expression levels using two different housekeeping genes confirmed that *OsUBC7* mRNA abundance increased at the flowering compared to the seedling stage, with log2 fold changes of 3.24 ± 0.20 and 3.94 ± 0.78 in *indica* and *japonica* accessions, respectively (Figure 2E,F; Supplementary Table S4).

To determine whether *OsUBC7* has a distinct expression pattern from other *RAD6*-type genes, we analyzed expression levels of two closely related paralogs, *OsUBC8* and *OsUBC9*. Normalized gene expression levels using two different housekeeping genes showed that compared to the seedling stage, *OsUBC8* mRNA levels were reduced at the flowering stage, with log2 fold changes of −1.10 ± 0.02 and −1.34 ± 0.56 in *indica* and *japonica* accessions, respectively, while *OsUBC9* mRNA levels did not change much at the flowering stage, with log2 fold changes of 0.64 ± 0.33 and 0.75 ± 0.03 in *indica* and *japonica* accessions, respectively (Figure 2E,F; Supplementary Table S4). Therefore, *OsUBC7* had a distinct temporal gene expression pattern: while it was strongly upregulated at the flowering stage in both *indica* and *japonica* rice accessions, the other two *RAD6* paralogs, *OsUBC8* and *OsUBC9*, were either downregulated or showed little expression change at the flowering compared to the seedling stage. These findings suggested that compared to its paralogs, *OsUBC7* may play a specific regulatory role during the flowering stage and when upregulated in response to cold stress.

### 3.11. Overexpression of OsUBC7 Tends to Improve Yield-Related Traits in Rice

Because *OsUBC7* is upregulated at the flowering stage, we investigated whether OE of the gene might affect plant yield-related traits, such as total seed number, number of seeds per panicle, and fertility. Averaging results from three generations of two *OsUBC7* OE lines showed that the OE lines had higher average seed yields than their segregated WT plants. Similarly, the two OE lines had increased seeds per panicle than WT plants. Additionally, OE lines had higher fertility scores than WT plants (Table 1). Due to large standard deviations of some samples, the trend was not significant; however, the results showed that both OE lines consistently outperformed their segregated WT plants for those yield-related phenotypes, suggesting that OE of *OsUBC7* might boost seed yield traits across at least three generations.

### 3.12. Overexpression of OsUBC7 Does Not Alter Flowering Time

Because *OsUBC7* is upregulated at the flowering stage, we investigated whether OE of *OsUBC7* affected flowering time of transgenic rice lines. No significant differences in flowering time between *OsUBC7* OE lines and segregated WT plants were detected. On average, all growth chamber-grown OE and WT lines flowered 98–100 days after germination (Table 1). Thus, *OsUBC7* is not a new flowering time gene.

### 3.13. Overexpression of OsUBC7 Increases Flag Leaf Length, Width, and Dry Weight

**Figure 7:**
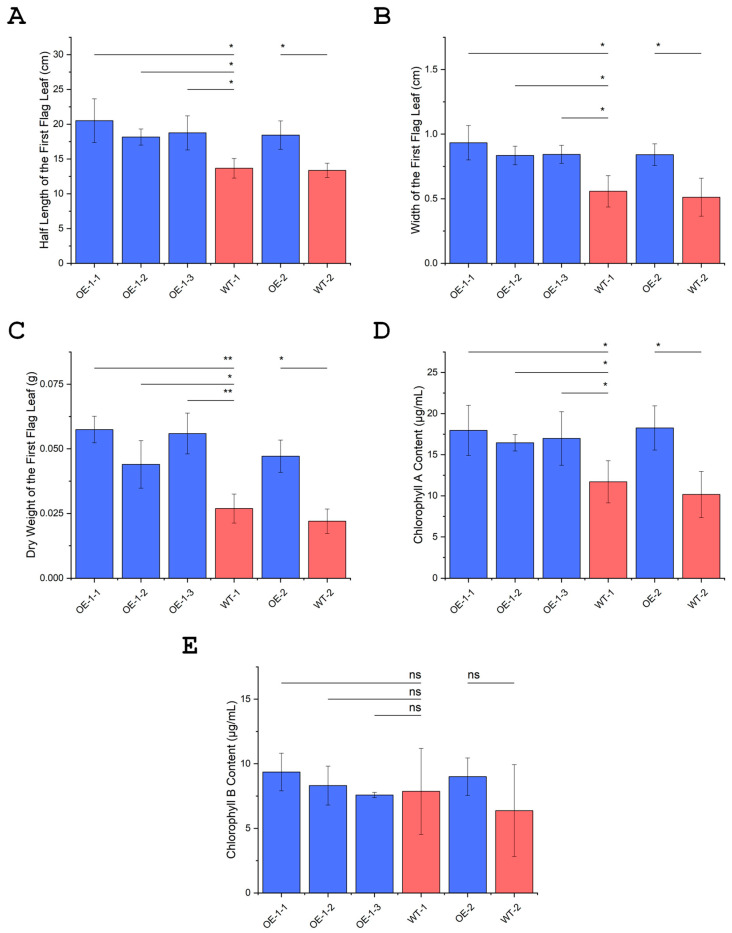
Architecture of the first flag leaf and chlorophyll content of *OsUBC7* overexpressing (OE) and wild-type (WT) lines. (**A**) Half-length of the first flag leaf, from the leaf tip to the middle. (**B**) Width of the first flag leaf. (**C**) Dry weight of the first flag leaf. (**D**) Total chlorophyll A content extracted from the entire flag leaf. (**E**) Total chlorophyll B content extracted from the entire flag leaf. *p*-values for Two-Way ANOVA: (*) < 0.05; (**) < 0.01; (ns) no significance.

Because *OsUBC7* OE lines were generally taller than segregated WT plants (Supplementary Figure S1E), we compared the architecture of their mature plant tissues by measuring the length, width, and dry weight of the flag leaf subtending the first panicle. OE lines had significantly longer, wider, and heavier flag leaves than WT plants (*p* < 0.05) (Figure 7A–C). Overall, flag leaves of OE lines were 1.40-fold longer, 1.61-fold wider, and 2.09-fold heavier than those of WT plants. These results indicated that OE of *OsUBC7* significantly (*p* < 0.05) alters plant architecture at the flowering stage, leading to longer, wider, and heavier flag leaves compared to WT plants (Supplementary Figure S1F).

### 3.14. Overexpression of OsUBC7 Leads to an Increase in Chlorophyll Content in Flag Leaves

Since OE of *OsUBC7* affected soluble sugar content and flag leaf architecture, we investigated whether OE also had an effect of the leaf chlorophyll content. OE lines had a significantly higher content of chlorophyll A than segregated WT lines (Figure 7D; *p* < 0.05). On the other hand, OE lines had a similar chlorophyll B content as WT plants (Figure 7E). This showed that OE of *OsUBC7* significantly increased chlorophyll A but not chlorophyll B content in flag leaves, possibly leading to enhanced photosynthetic capacity at this stage.

### 3.15. OsUBC7 Interacts with the E3 Protein OsRFPH2-12 and the RING Finger Domain of the Two E3 Proteins OsHUB1 and OsHUB2

**Figure 8:**
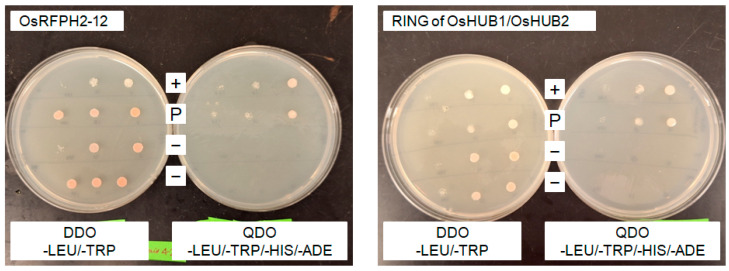
Yeast two-hybrid assay showing interaction between Gal4-DNA binding domain (BD)-OsUBC7 and Gal4-Activation domain (AD)-E3 ligase fusions of OsRFPH2-12 (**Left**) and OsHUB1/HUB2 (**Right**; RING finger domain only). Positive interactions are shown by robust colony growth on quadruple dropout (QDO) selective plates, as seen for OsUBC7::RFPH2-12 and OsUBC7::OsHUB1/HUB2 in rows labeled “P”. A positive control showing interaction between a Gal4-BD-murine P53 fusion (bait) and a Gal4-AD-SV40 large T-antigen fusion (prey) is seen in rows labeled “+”. Negative controls of yeast transformed with the OsUBC7 bait and an empty prey vector (and vice versa) have no growth on QDO selective plates, as shown in rows labeled “–”. Bait and prey plasmids have robust growth on double dropout (DDO) selective plates.

Because E2 ubiquitin-conjugating enzymes form complexes with E3 ligases and target proteins, we interrogated the String database [https://string-db.org/ (accessed on 19 November 2020] to identify potential UBC7-interacting proteins. From this we selected an E3 ligase expressed during the flowering stage, *OsRFPH2-12*, and two histone E3 ligases potentially involved in cell cycle regulation, *OsHUB1* and *OsHUB2*. To determine whether OsUBC7 physically interacted with those E3 ligases, we performed yeast two-hybrid assays, indicating that OsUBC7 interacted with the full-length OsRFPH2-12 protein and the RING finger domains of OsHUB1/HUB2, as evidenced by robust growth on quadruple selective media lacking adenine, histidine, tryptophan, and leucine (Figure 8). These findings suggested that OsUBC7 may act as an E2 ubiquitin-conjugating enzyme in concert with developmental and cell cycle stage-specific E3 ligases to regulate protein abundance and/or protein function.

Since the protein sequences of OsUBC7 and its Arabidopsis homolog AtUBC2 are highly conserved by sharing 145/152 identical and 151/152 positive amino acids, we performed an unbiased yeast two-hybrid screen with OsUBC7 as bait and a commercial Arabidopsis cDNA library made from all tissues and developmental stages as prey. This screen yielded E3 ligases, cell cycle-dependent kinases, other kinases, transcription factors, enzymes, a hydroxyproline-rich glycoprotein, and a GCN5-type histone-modifying acetyltransferase (Supplementary Table S5). These proteins are potential OsUBC7 targets that might have roles in cold stress tolerance response mechanisms, and rice homologs of those proteins merit future investigations.

### 3.16. Overexpression of OsUBC7 Tends to Increase Germination Rates of Arabidopsis Seeds Under High Salinity Stress

Because abiotic stress response mechanisms often converge through a network of coordinated biochemical and molecular pathways, we investigated whether *OsUBC7* could improve high salinity stress tolerance by performing germination assays with seeds from homozygous *OsUBC7* OE Arabidopsis lines and WT control plants. We found significant differences in germination rates between *OsUBC7* OE and WT lines over the two-week period on 50 and 100 mM NaCl (Supplementary Table S6). This suggested that *OsUBC7* may play a role in different abiotic stress tolerance response mechanisms.

## 4. Discussion

### 4.1. OsUBC7 May Mediate Cold Stress Tolerance Responses Through Sugar Metabolism and Growth Regulation

The identification of *OsUBC7* as a candidate gene associated with a low-temperature seedling survivability (LTSS) trait QTL in rice [2] is supported by its differential expression patterns during cold induction and single-nucleotide polymorphisms (SNPs) between chilling-tolerant and chilling-sensitive accessions (Figure 1A and Figure 2; Supplementary Table S3). The significant correlation between *OsUBC7* haplotypes and LTSS scores (Figure 1C) further emphasizes its potential in modulating cold stress tolerance responses in rice.

*OsUBC7* overexpression (OE) lines overall had higher LTSS scores than wild-type (WT) plants after cold treatment, which agrees with the hypothesis that upregulation of this gene during cold helps boost plant resilience to low-temperature stress in both rice and Arabidopsis (Figure 3 and Figure 4A,B; Supplementary Figure S1A–D). However, the similar LTSS scores between OE and WT lines after extended cold exposure suggests that *OsUBC7*-mediated stress tolerance mechanisms are limited to early timepoints of stress exposure.

Our findings suggest that a potential mechanism involved in boosting cold stress resilience in *OsUBC7* OE lines might be the higher growth rate in OE than WT lines before and after cold stress (Figure 4C,D), which may be critical for successful recovery and enhanced survivability. This might occur through the restoration of cellular homeostasis and regulation of new cell growth, particularly in leaf cells (Figure 4E), which are critical for photosynthesis and metabolic activity [6,34,35]. The increased growth rate and higher number of cells per unit leaf area (Figure 6A,B) might contribute to the elevated levels of soluble sugars observed in *OsUBC7* OE lines before, during, and after cold or freezing stress (Figure 5A,B). Boosting of cold tolerance via growth regulation has been previously observed. For example, RNAi-mediated knockdown of *OsOFP6* reduced the number of leaf cells, which reduced the percent of starch-filled pollen grains under cold treatment [36]. OE of *OsCYP19-4* promoted an increase in tiller and spike numbers and grain weight, which correlated with enhanced cold tolerance [37]. OE of *OsRAN2*, an essential protein for mitosis, enhanced cold tolerance by promoting export of intranuclear tubulin and maintaining cell division under cold stress and recovery [38]. OE of *OsMYB3R-2* helped with cell cycle progression during chilling stress [39]. These findings agree with our interpretation that OE of *OsUBC7* might boost LTSS scores by increasing the growth rate and soluble sugar content of transgenic plants.

The observed increase in soluble sugar levels before stress may serve as a critical energy source for *OsUBC7* OE plants during cold stress and subsequent recovery periods, facilitating essential metabolic processes required for recovery and survival (Figure 4C,D and Figure 5A,B) [12,40,41]. Interestingly, the increase in soluble sugar content peaked during cold or freezing stress for *OsUBC7* OE plants, while it peaked one day after recovery in WT plants (Figure 5A,B), indicating a dynamic regulation of genes, proteins, and metabolites throughout the stress-response period [42,43,44]. In agreement with this, OE of *OsSUT1-5* increased total sucrose levels prior to chilling treatment and helped with the plant phloem loading of sucrose [45], and OE of *OsTERF2* increased accumulation of soluble sugar and other osmotic substances as well as overall chlorophyll content [46]. Moreover, OE of *OsCIPK03* and *OsCIPK12* significantly increased soluble sugar contents and improved cold stress tolerance [47], and OE of the *japonica* allele of *OsbZIP73* increased soluble sugar contents under chilling stress [48]. Thus, in agreement with these studies, elevated soluble sugar contents in *OsUBC7* OE lines suggests a potential role in regulating cold tolerance by modulating soluble sugar-mediated metabolic processes.

Work with the Arabidopsis homolog of *OsUBC7* showed that knocking out *AtUBC2* significantly reduced histone H2B mono-ubiquitination, leading to gene- and protein-specific changes in the patterns of histone ubiquitination and methylation [49]. *AtUBC2* moreover was shown to have roles in regulating protein homeostasis and growth of Arabidopsis plants under high salinity conditions [20]. Furthermore, overexpression of the soybean *RAD6* homolog *GmUBC9* increased drought resistance and affected flowering time via histone H2B mono-ubiquitination in both Arabidopsis and soybean plants [21]. In agreement, our germination assays showed that *OsUBC7* OE Arabidopsis lines maintained nearly 100% germination rates compared to WT under high salinity stress (Supplementary Table S6), indicating that *OsUBC7* is involved in other abiotic stress tolerance mechanisms.

Interestingly, an increase in soluble sugar content has been previously linked to maintenance of plasma membrane integrity [1,4,11,12]; however, our *OsUBC7* OE lines did not have lower electrolyte leakage or lipid peroxidation levels than wild-type plants after cold stress (Figure 5C–E). One explanation for this is that the observed increase in soluble sugar in *OsUBC7* OE plants provided energy for other metabolic processes including cell cycle progression, leading to higher number of cells per leaf area and better recovery growth and development after cold stress exposure.

### 4.2. Dynamic Expression of OsUBC7 in Response to Cold Stress and Flowering

We show here that mRNA abundance of *OsUBC7* increases in response to prolonged cold treatment, but the extent of upregulation was higher in cold-tolerant *japonica* than cold-sensitive *indica* (Figure 2A–D; Supplementary Table S3). This differential response may contribute, at least in part, to the higher cold tolerance potential of *japonica* than *indica* accessions [1,2,10]. However, both subspecies probably employ the E2-conjugating enzyme function of OsUBC7 as part of a conserved cold stress tolerance response mechanism that might positively affect protein homeostasis [50,51], which involves maintaining a balanced and functional proteome within cells, ensuring proper protein folding, localization, and function [52,53]. Interfering with the proteostasis mechanism can have both positive and negative effects [22,54]. For example, OE of the two E3 ligase-encoding genes *OsPUB2/PUB3* or *OsDIRP1* boosted cold stress tolerance and chlorophyll content of transgenic rice [23,24]. Conversely, OE of the E3 ligase-encoding gene *OsATL38* led to turnover of OsGF14, a positive regulator of cold tolerance, thus, negatively affecting cold stress tolerance in rice [55], while knocking down the stress inducible E3 ligase-encoding gene *OsSRFP1* improved cold stress tolerance [56]. Taken together, upregulating genes such as *OsUBC7* in both *indica* and *japonica* accessions may be beneficial for maintaining protein homeostasis during cold stress and during recovery after stress exposure [50,51,52,53].

Based on its expression profile, *OsUBC7* may have a predominant function during the flowering stage of rice development, because compared to its *RAD6* paralogs *OsUBC8* and *OsUBC9*, it was specifically upregulated at that developmental stage (Figure 2E,F; Supplementary Table S4). These expression profiles agree with RNA-seq and microarray results deposited into publicly available databases such as RiceXPro, the Rice RNA-seq Database, and the Rice Genome Annotation Project [14,15,19]. To the best of our knowledge, we show here for the first time that the E2-conjugating enzyme encoding gene *OsUBC7* has an additional function as a cold stress-upregulated gene, which implies that the OsUBC7 protein might interact with different E3 ligase partners and target proteins at the flowering stage than during cold induction at the young seedling stage.

### 4.3. A Role for OsUBC7 in Promoting Rice Cell Proliferation

As a RAD6-type protein, OsUBC7 has a predicted role as a checkpoint protein in the cell cycle [14,15], suggesting a role in regulating cell proliferation and expansion. This prediction is supported by the increased growth rates we observed in *OsUBC7* OE seedlings (Figure 2C–F and Figure 5A–C). The increased growth rate promoted by *OsUBC7* OE was mediated by an increase in cell density per unit leaf area rather than cell elongation (Figure 4B), suggesting that *OsUBC7* may primarily regulate cell proliferation rather than cell elongation. From the very well-studied functions of RAD6 in yeast as a protein involved in DNA repair and cell cycle regulation [17,18,57], we can draw further parallels to the potential molecular mechanisms underlying OsUBC7-mediated growth regulation. Because yeast RAD6 is upregulated during the G1/S and S/G2 phase transitions to serve as a checkpoint protein during cell division [16,57], OsUBC7 may similarly play a role in modulating cell cycle progression and growth dynamics. The longer cells stay in G1 the larger they become [58,59]; therefore, it is possible that OsUBC7 may shorten the cell cycle by speeding up the transition from G1 to S phase, thus, making the cell narrower and increasing cell density over time. In support of this, OE of *RAD6* in human cells led to a quicker transition from the G1 to S phase and to increased cell proliferation [13]. Taken together, *OsUBC7* might have a similar role as its yeast homolog *RAD6* in regulating cell cycle progression and growth dynamics in rice, possibly by promoting a quicker transition from the G1 to S phase, leading to increased cell density and proliferation.

### 4.4. A Role for OsUBC7 in Yield Enhancement and Flowering Regulation of Rice

Our analysis of yield-related traits revealed a strong trend toward increases in seed yield, seeds per panicle, and fertility in *OsUBC7* OE compared to segregated WT lines (Table 1). Since *OsUBC7* is located on a 3.5 Mb segment of chromosome 7 that overlaps with several yield-related trait QTL such as number of grains per panicle (*gp7a*), 1000-grain weight (*gw7*), yield per plant (*yd7a*), and number of tillers per plant (*tp7b*) [60,61]; it is possible that *OsUBC7* is a candidate gene for those traits, as supported by our observations. However, this trend toward higher yields was not due to changes in flowering time, because *OsUBC7* OE and WT lines had similar heading dates (Table 1). Thus, *OsUBC7* is not a novel flowering time gene. This result agrees with flowering time studies in Arabidopsis where it was shown that although the *OsUBC7* homolog *AtUBC2* was involved in histone H2B mono-ubiquitination in the chromatin region of the flowering repressor gene *FLOWERING LOCUS C* [62,63], OE of *AtUBC2* did not affect flowering time [49].

Additionally, OE of *OsUBC7* effected flag leaf morphology and physiology, leading to longer, wider, and heavier flag leaves compared to segregated WT plants (Figure 7A–C; Supplementary Figure S1F). This suggests that *OsUBC7*, annotated as a cell division checkpoint protein-encoding gene [15], may play a role in regulating leaf growth and development during the flowering stage, where it has the highest expression levels. Interestingly, OE of *OsUBC7* also resulted in higher chlorophyll A content in the flag leaves compared to WT plants (Figure 7D). Together with a higher cell density, the higher chlorophyll content most likely led to enhanced photosynthetic activity and potentially improved plant vigor in *OsUBC7* OE lines [64,65,66,67].

### 4.5. Interaction of OsUBC7 with E3 Type RING Finger Domain and Potential Target Proteins

Our yeast two-hybrid assay showed that OsUBC7 physically interacts with OsRFPH2-12, an E3 ligase co-expressed with OsUBC7 during flowering in rice, and with the RING finger domain of the more broadly expressed E3 ligases OsHUB1/2 (Figure 8), indicating that OsUBC7 has different E3 partners that may have different roles in rice development and stress tolerance response mechanisms. OsHUB1 and OsHUB2 are involved in mono-ubiquitinating histone H2B [68,69,70], a key modification that regulates chromatin structure and gene expression [25,71]. This histone modification affects expression of genes involved in both normal growth and abiotic stress responses [21,23,25,71], and most likely facilitates upregulation of cold responsive genes such as those coding for CBF/DREB1 transcription factors that are early regulators of the cold stress tolerance responses [72,73] by activating COR genes that help stabilize cell membranes, protect cellular proteins, and maintain osmotic balance under low-temperature stress [74,75].

In addition to potentially regulating gene expression, OsUBC7 likely contributes to protein turnover through its interaction with the E3 ligase OsRFPH2-12, which may target key regulatory proteins for degradation during the flowering stage of rice development [76]. During both developmental switches and cold stress responses, cells must rapidly degrade obsolete or damaged proteins, as well as negative regulators of stress response pathways to enable the activation of protective mechanisms [77,78,79]. Flowering is a highly regulated developmental event, influenced by environmental cues such as day length and temperature [49,79,80], and timely protein degradation of specific inhibitors is required to ensure that flowering signals can proceed [49,63,77]. Perhaps the main function of OsUBC7 during rice development is degradation of proteins that inhibit flowering by interacting with developmental stage-specific E3 ligases such as OsRFPH2-12, while it assumes a specialized function during cold stress tolerance responses by complexing with different E3 partners such as OsHUB1 or OsHUB2 to mono-ubiquitinate histones. A better understanding of how OsUBC7 integrates these different functions will provide valuable insights into potential strategies for enhancing cold tolerance and perhaps yield in rice, such as via targeted manipulation of ubiquitination pathways to optimize stress response and growth. Identification of additional developmental stage or cold stress response E3 partners and protein targets will help with this task. We have already obtained valuable information using an unbiased yeast-two hybrid screen (Supplementary Table S5), and future studies on rice homologs of those OsUBC7-interacting Arabidopsis proteins are merited.

## 5. Conclusions

The present study revealed multiple roles for *OsUBC7* in the rice cold temperature stress tolerance response, highlighting its involvement in orchestrating sugar metabolism and growth regulation. Although the main function of *OsUBC7* in rice most likely occurs during the flowering stage, we uncovered via genomics, differential gene expression, and overexpression analyses an additional function for *OsUBC7* as a cold-induced gene, indicating that it has a potential for enhancing plant fitness in response to low-temperature and possibly other abiotic stress. We propose that an interaction of OsUBC7 with the histone H2B mono-ubiquitinating E3 ligases OsHUB1 and OsHUB2 has a role in the early cold stress tolerance response mechanisms by activating positive regulators of the stress response and metabolic processes leading to higher amounts of soluble sugars providing resources for growth and development during stress recovery. The present study contributes to the recent interest in exploring the involvement of *OsUBC* genes in low-temperature germination (via ABA signaling; [26]), root development (via auxin signaling; [81]), pathogen response [82,83], and regulation of plant height and yield [83,84].

## Figures and Tables

**Table 1:** Yield-related trait phenotypes of *OsUBC7* overexpression (OE) and segregated wild-type (WT) lines in the Kasalath (*aus*) accession.

Yield-Related Phenotype	Line	Data from Three Generations	*p*-Value (Two-Way ANOVA)
Total Seeds per Plant	OE-1	80.3 ± 33.7	0.124
	WT-1	30.7 ± 3.3	
	OE-2	66.9 ± 16.5	0.072
	WT-2	36.3 ± 14.1	
Number of Seeds per Panicle	OE-1	34.4 ± 12.7	0.278
	WT-1	23.5 ± 6.9	
	OE-2	32.2 ± 17.5	0.274
	WT-2	17.0 ± 9.2	
Fertility (%)	OE-1	86.4 ± 1.0	0.080
	WT-1	77.0 ± 4.1	
	OE-2	83.7 ± 5.1	0.163
	WT-2	62.1 ± 17.8	
Flowering Time (No. of Days to heading)	OE-1-1	101.2 ± 1.9	0.684
	OE-1-2	100.3 ± 2.8	0.933
	OE-1-3	102.2 ± 5.4	0.648
	OE-2	100.3 ± 1.9	0.375
	WT-1	100.5 ± 2.2	
	WT-2	98.5 ± 2.5	

## Data Availability

The original contributions presented in this study are included in the article/Supplementary Material. Further inquiries can be directed to the corresponding author.

## Supplementary Materials

The following supporting information can be downloaded at: https://www.mdpi.com/article/10.3390/genes16010066/s1, Table S1: Sequences of primers used in the present study; Table S2: qPCR measurements of mRNA levels in 2-week-old seedlings containing *OsUBC7* overexpression (OE) constructs in the Kasalath (*aus* subgroup) genetic background compared to their respective wild-type (WT) plants under warm growth conditions; Table S3: mRNA abundance of *OsUBC7* in *aus* Kasalath and *temperate japonica* Krasnodarskij 3352 (Krasnodars.) in 2-week-old seedlings under warm condition (0 h) and after different periods of cold temperature exposure (10 °C for Kasalath; 4 °C for Krasnodars.); Table S4: Expression levels of *OsUBC7*, *OsUBC8*, *OsUBC9* in *Indica* Kasalath (*aus*) and *Japonica* Krasnodarskij 3352 (Krasnodars.; *temperate japonica*) in 2-week-old seedlings and flag leaves at the flowering stage; Table S5: Arabidopsis genes corresponding to expressed sequence tags (EST) isolated from a yeast two-hybrid screen using OsUBC7 as bait; Table S6: Germination rates of Arabidopsis seeds from Col-0 wild-type (WT) plants and *OsUBC7* overexpression (OE) lines under different NaCl concentrations (0, 50, 100 mM); Figure S1: Phenotypes of *OsUBC7* overexpression (OE) Arabidopsis and rice plants.
