# Supplementary material for: The RAD6-like Ubiquitin Conjugase Gene OsUBC7 Has a Positive Role in the Early Cold Stress Tolerance Response of Rice
Source: Genes (Basel). 2025 Jan 8;16(1):66. doi: 10.3390/genes16010066 (PMC11764743; doi:10.3390/genes16010066)
Supplement: Supplementary file 1 [file genes-16-00066-s001.zip › genes-3352921-supplementary.pdf]

genes

MDPI

Article

The *RAD6*-like Ubiquitin Conjugase Gene *OsUBC7* Has a Positive Role in the Early Cold Stress Tolerance Response of Rice

Huy Phan and Michael Schläppi *

Department of Biological Sciences, Marquette University, Milwaukee, WI 53233, USA; huy.phan@marquette.edu
* Correspondence: michael.schlappi@marquette.edu; Tel.: +1-414-288-1480

Abstract: Background/Objectives: Cold stress poses a significant threat to Asian rice cultivation, disrupting important physiological processes crucial for seedling establishment and overall plant growth. It is, thus, crucial to elucidate genetic pathways involved in cold stress tolerance response mechanisms. Methods: We mapped *OsUBC7*, a *Radiation-sensitive 6* (*RAD6*)-type homolog of rice, to a low-temperature seedling survivability (LTSS) QTL and used genomics, molecular genetics, and physiological assays to assess its role in plant resilience against low-temperature stress. Results: *OsUBC7* is cold responsive and has higher expression levels in cold-tolerant *japonica* than cold-sensitive *indica*. Overexpression of *OsUBC7* enhances LTSS of *indica* and freezing tolerance of Arabidopsis, increases levels of soluble sugars and chlorophyll A, boosts leaf development after cold exposure, and increases leaf cell numbers and plants size, but it does not affect membrane stability after cold stress exposure. Additionally, *OsUBC7* has a positive role for germinability in the presence of salt and for flowering and yield-related traits. The OsUBC7 protein physically interacts with the developmental stage-specific and histone-modifying E3 ligases OsRFPH2-12 and OsHUB1/2, respectively, and potential target genes such as cell cycle dependent kinases were identified. Conclusions: *OsUBC7* might contribute to cold resilience by regulating sugar metabolism to provide energy for promoting cellular homeostasis restoration after cold stress exposure via new cell growth, particularly in leaf cells crucial for photosynthesis and metabolic activity, possibly by interacting with cell cycle regulating proteins. Overall, the present study suggests that *OsUBC7* may be involved in plant development, reproduction, and stress adaptation, and contributes to a deeper understanding of rice plant cold stress tolerance response mechanisms. *OsUBC7* may be a promising candidate for improving crop productivity and resilience to stressful environments.

Keywords: chilling stress; LTSS; low-temperature stress recovery; *Oryza sativa* L.

Academic Editors: Bing Yang and Sheng Zhang

Received: 19 November 2024
Revised: 11 December 2024
Accepted: 6 January 2025
Published: 8 January 2025

Citation: Phan, H.; Schläppi, M. The *RAD6*-like Ubiquitin Conjugase Gene *OsUBC7* Has a Positive Role in the Early Cold Stress Tolerance Response of Rice. *Genes* **2025**, *16*, 66. https://doi.org/10.3390/genes16010066

Copyright: © 2025 by the authors. Licensee MDPI, Basel, Switzerland. This article is an open access article distributed under the terms and conditions of the Creative Commons Attribution (CC BY) license (https://creativecommons.org/licenses/by/4.0/).

Supplementary Materials

Supplementary Table S1. Sequences of primers used in the present study to investigate the molecular function of the rice *RAD6*-like gene *OsUBC7*.

**Primer name**	**Sequence**
OsUBC7_F (BamHI for cloning)	GGATCCGGGAGATCGAAACCCTAGC
OsUBC7_R (EcoRI for cloning)	GAATTCTTTCTGGCATGGTGGATAC
OsUBC7_F4	CGACTGTTCGGTTTGTTT
OsUBC7_R4	TCATATATAGGGCTCCACTG
18S_rRNA_F	CTACGTCCCTGCCCTTTGTACA
18S_rRNA_R	ACACTTCACCGGACCATTCAA
OsACT1_F1	GCGTGATCTCACTGATTAC
OsACT1_R1	CTTGGCAGTCTCCATTTC
OsUBC32_F2	CAAGTTCGCTTCTTGAC
OsUBC32_R2	CTGTCTGGTTCAGGATG
OsUBQ5_F	ACCACTTCGACCGCCACTACT
OsUBQ5_R	ACGCCTAAGCCTGCTGGTT
OsUBC8_F1	GCTGCTATACTCACATCCATCC
OsUBC8_R1	GCTCCACTATCTCACGAACTTT
OsUBC9_F1	CGGTTTGTCTCAAGGATGTTTC
OsUBC9_R1	AGGGACTGGATAGAGGTCAATA

Supplementary Table S2. qPCR measurements of mRNA levels in 2-week-old seedlings containing *OsUBC7* overexpression (OE) constructs in the Kasalath (*aus* subgroup) genetic background compared to their respective wild-type (WT) plants under warm growth conditions. 18S rRNA and *OsACT1* were used to normalize expressions levels.

House-Keeping Genes	Rice Lines	Normalized Expression	Log2 Fold-Change
18S ribosomal RNA	OE-1-1	1.16E-03±9.20E-04	1.516±0.678
	OE-1-2	1.47E-03±4.80E-04	2.036±0.322
	OE-1-3	2.52E-03±3.50E-03	2.151±2.088
	OE-2	1.48E-03±1.41E-03	1.660±1.144
	WT-1	4.10E-04±2.60E-04	0
	WT-2	7.90E-04±1.01E-03	0
OsACT1	OE-1-1	18.69±12.87	2.205±0.422
	OE-1-2	25.80±15.88	2.658±0.411
	OE-1-3	16.48±12.42	2.007±0.526
	OE-2	14.52±9.51	1.960±0.474
	WT-1	3.86±1.67	0
	WT-2	3.68±2.11	0

Supplementary Table S3. mRNA abundance of *OsUBC7* in *aus* Kasalath and *temperate japonica* Krasnodarskij 3352 (Krasnodars.) in 2-week-old seedlings under warm condition (0 h) and after different periods of cold temperature exposure (10 °C for Kasalath; 4 °C for Krasnodars.). Four house-keeping genes were used to normalize mRNA abundance: 18S ribosomal RNA, *OsACT1*, *OsUBC32*, and *OsUBQ5*.

House-Keeping Gene	Hrs of Exposure	Normalized mRNA Abundance		Log2 Fold-Change	
		Kasalath	Krasnodars.	Kasalath	Krasnodars.
**18S ribosomal RNA**	0	2.09E-03 ± 1.20E-04	1.63E-03 ± 2.90E-04	0	0
	12	3.13E-03 ± 4.06E-03	2.30E-03 ± 6.20E-04	0.585	0.496
	24	3.12E-03 ± 2.80E-04	3.86E-03 ± 1.57E-03	0.575	1.245
	36	4.11E-03 ± 1.25E-03	1.10E-02 ± 2.55E-03	0.978	2.751
	48	4.35E-03 ± 1.26E-03	7.51E-03 ± 2.56E-03	1.057	2.205
OsACT1	0	1.13E-01 ± 8.10E-02	7.70E-02 ± 1.30E-02	0	0
	12	1.54E-01 ± 4.90E-02	1.58E-01 ± 3.60E-02	0.444	1.029
	24	2.16E-01 ± 9.20E-02	3.81E-01 ± 1.92E-01	0.941	2.305
	36	5.46E-01 ± 1.31E-01	6.05E-01 ± 4.67E-01	2.275	2.973
	48	6.26E-01 ± 2.92E-01	7.89E-01 ± 1.38E-01	2.470	3.356
OsUBC32	0	5.15E-02 ± 1.10E-02	3.00E-02 ± 1.07E-02	0	0
	12	6.87E-02 ± 2.26E-02	5.20E-02 ± 1.48E-02	0.411	0.791
	24	1.90E-01 ± 3.45E-02	2.48E-01 ± 8.90E-02	1.880	3.046
	36	2.41E-01 ± 3.00E-02	3.04E-01 ± 1.50E-01	2.227	3.338
	48	2.18E-01 ± 9.81E-02	3.03E-01 ± 1.48E-01	2.084	3.333
OsUBQ5	0	5.22E-02 ± 1.97E-02	4.23E-02 ± 2.55E-02	0	0
	12	2.22E-01 ± 8.00E-03	1.40E-01 ± 3.95E-02	2.091	1.731
	24	2.76E-01 ± 1.11E-01	3.28E-01 ± 1.09E-01	2.403	2.954

	36	2.76E-01 ± 6.04E-02	4.96E-01 ± 6.11E-02	2.403	3.551
	48	3.55E-01 ± 1.31E-01	1.07E+00 ± 2.18E-01	2.766	4.659

Supplementary Table S4. Expression levels of *OsUBC7*, *OsUBC8*, *OsUBC9* in *Indica* Kasalath (*aus*) and *Japonica* Krasnodarskij 3352 (Krasnodars.; *temperate japonica*) in 2-week-old seedlings and flag leaves at the flowering stage. Two house-keeping genes were used: 18S ribosomal RNA, *OsACT1*.

House-Keeping Genes	Target Gene and Development Stage	Relative mRNA Abundance		Log2 Fold-Change	
		Kasalath	Krasnodars.	Kasalath	Krasnodars.
	OsUBC7				
**18S ribosomal RNA**	2-week-old	2.09E-03 ± 1.20E-04	1.63E-03 ± 2.90E-04	0	0
	Flowering	1.78E-02 ± 9.07E-03	1.71E-02 ± 4.60E-03	3.093	3.392
OsACT1	2-week-old	1.13E-01 ± 8.10E-02	7.71E-02 ± 1.34E-02	0	0
	Flowering	1.78E+00 ± 3.88E-01	1.74E+00 ± 4.30E-01	3.379	4.493
	OsUBC8				
**18S ribosomal RNA**	2-week-old	1.22E-02 ± 4.81E-03	1.36E-02 ± 5.92E-03	0	0
	Flowering	5.55E-03 ± 1.81E-03	4.01E-03 ± 8.80E-04	-1.120	-1.737
OsACT1	2-week-old	1.41E+00 ± 3.49E-01	1.20E+00 ± 2.59E-01	0	0
	Flowering	6.58E-01 ± 1.66E-01	6.23E-01 ± 1.00E-01	-1.089	-0.943
	OsUBC9				
**18S ribosomal RNA**	2-week-old	5.49E-02 ± 2.12E-02	5.44E-02 ± 1.21E-02	0	0
	Flowering	1.00E-01 ± 1.96E-02	9.04E-02 ± 2.59E-02	0.872	0.731
OsACT1	2-week-old	1.69E+01 ± 9.10E+00	1.13E+01 ± 1.07E+00	0	0
	Flowering	2.23E+01 ± 8.68E+00	1.94E+01 ± 1.89E+00	0.401	0.774

Supplementary Table S5. Arabidopsis genes corresponding to expressed sequence tags (EST) isolated from a yeast two-hybrid screen using OsUBC7 as bait. No. of clones indicates how many times the same or similar ESTs were recovered. No of homologs in *Oryza sativa* L. indicates how many homologs, including paralogs, are found in the rice genome.

Accession	Encoding Protein	No. of Clones	No. of Homologs in *Oryza sativa* L.
At1g74490	PBS1-like 29 protein kinase	1	2
At3g01085	Cyclin-dependent protein kinase	2	>2
At1g53400	Ubiquitin domain-containing protein	1	3
At4g34100	CER9 ubiquitin ligase	2	1
At1g01060	LHY myb-related transcription factor	2	6
At2g06025	GCN5-related N-acetyltransferase	1	1
At1g77330	ACC oxidase 5 (ethylene biosynthesis)	1	2
At1g04930	Hydroxyproline-rich glycoprotein (SIC)	2	1

Supplementary Table S6. Germination rates of Arabidopsis seeds from Col-0 wild-type (WT) plants and *OsUBC7* overexpression (OE) lines under different NaCl concentrations (0, 50, 100 mM).

NaCl Concentration	OE-1	Col-0 WT	Two-way ANOVA against 0 mM
	Normalized against 0 mM		
0 mM	70.0±5.3 (%) (Raw) 100.00 ± 0.00 (%) (Normalized)	86.4±4.2 (%) (Raw) 100.00 ± 0.00 (%) (Normalized)	
50 mM	72.4±5.9 % (Raw) 103.5±5.4 (%) (Normalized)	77.3±7.1 % (Raw) 89.5±5.3 (%) (Normalized)	0.03296 (Normalized)
100 mM	71.7±8.8 % (Raw) 102.5±9.4 (%) (Normalized)	58.6±5.8 % (Raw) 67.7±3.4 (%) (Normalized)	0.01503 (Normalized)

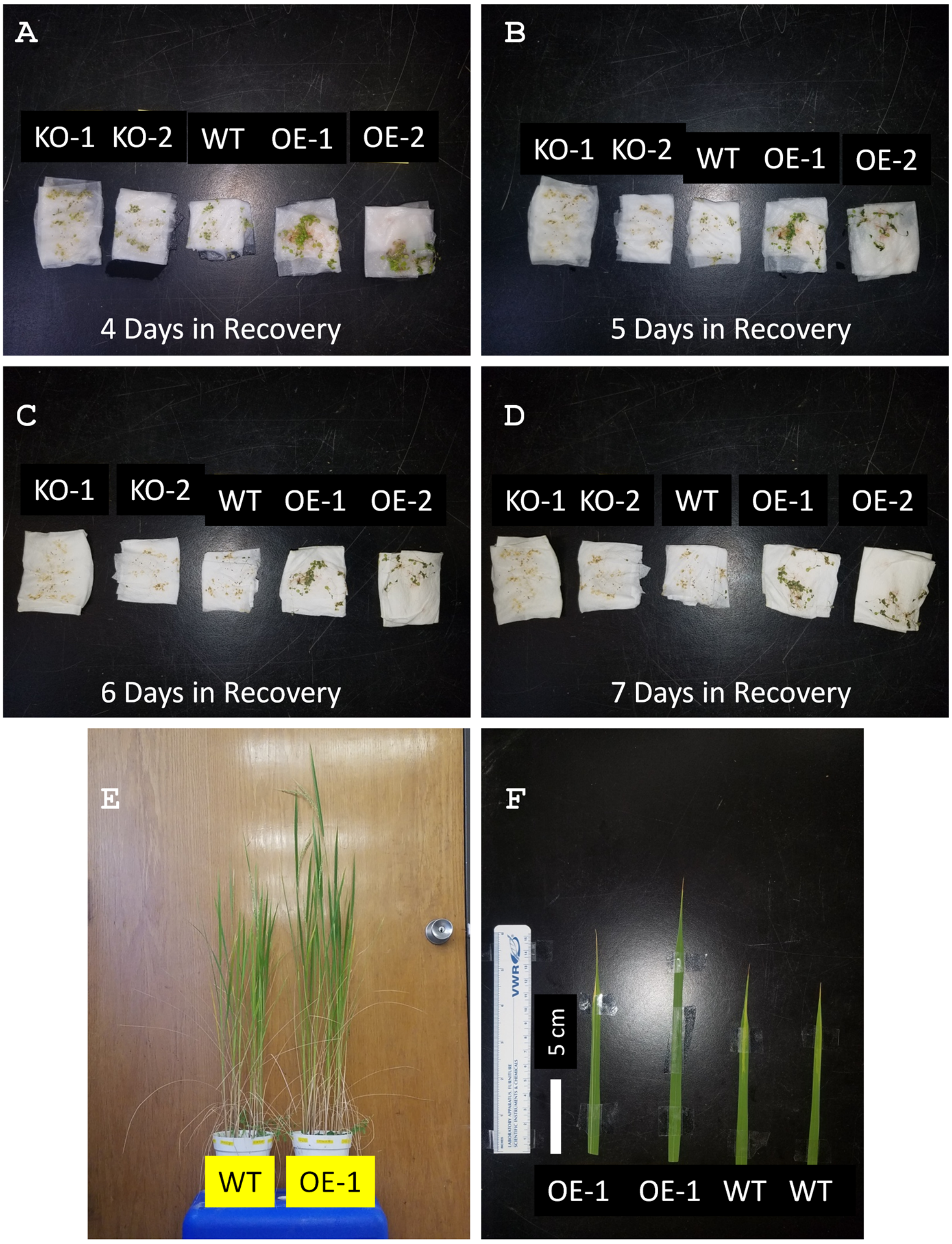

Supplementary Figure S1. Phenotypes of *OsUBC7* overexpression (OE) Arabidopsis and rice plants. (A-D) *OsUBC7* OE Arabidopsis, Col-0 wild-type (WT), and *AtUBC2* (*OsUBC7* homolog) knockout (KO) plants exposed to -3 °C and recovering over a 7-day period. Dead plants are bleached white, and surviving plants are green. (E) Height comparison of *OsUBC7* OE and Kasalath WT rice plants, 3 days after the first flag leaf emerged (6–8 plants per pot are shown). (F) Length and width comparison of the first flag leaf of *OsUBC7* OE and Kasalath WT rice plants.
